# Editorial: Special Issue “Emerging Sensor Technology in Agriculture”

**DOI:** 10.3390/s20143827

**Published:** 2020-07-09

**Authors:** Carlos Poblete-Echeverría, Sigfredo Fuentes

**Affiliations:** 1 Department of Viticulture and Oenology, Faculty of AgriSciences, Stellenbosch University, Private Bag X1, Matieland 7602, South Africa; cpe@sun.ac.za; 2 Digital Agriculture, Food and Wine Sciences Group, School of Agriculture and Food, Faculty of Veterinary and Agricultural Sciences, University of Melbourne, Melbourne, VIC 3010, Australia

Research and innovation activities in the area of sensor technology can accelerate the adoption of new and emerging digital tools in the agricultural sector by the implementation of precision farming practices such as remote sensing, operations, and real-time monitoring. The agricultural industry has been greatly affected by climate change; therefore, to be successful in overcoming these effects and remain competitive and sustainable in the market, there is the need to support research and application development of new and emerging sensor technologies and their applications in agriculture. A total of 13 papers were published in this Special Issue entitled: “Emerging Sensor Technology in Agriculture”, and the topics addressed include different emerging technologies with applications on ecosystems (grasslands) [1] and several agriculture crops such as peppers [2], apples [2,3], grapevines [2,4,5,6,7], cocoa trees [6], citrus [8], legumes [9], wheat and rice [10,11]. Two papers were also related to the use of remote sensing to detect forage quality [9], regions of interest of pigs [12], and pesticide droplet deposition [13] using machine learning.

In Rueda-Ayala et al. [1] an aerial (Unmanned aerial vehicle—UAV) and an on-ground (Kinect sensor—RGB-D (Depth camera)) methods were used to characterize grass ley fields (plant height, biomass, and volume) composed of different species mixtures, using digital grass models. In this study, both methods presented a good performance. From a comparison point of view, the authors took into consideration some basic economic and practical aspects of the methodologies. Hacking et al. [4] used a similar approach to determine yield in grapevines. Another UAV-based study investigated the effect of eddies formed at low altitude in wheat to estimate water status effectively and other physiological parameters in rice [11]. Yield estimation is a key topic in agriculture in general, and it is very relevant in viticulture since winegrowers need such information to manage several logistic aspects at the cellars. In Hacking et al. [4], 2D (RGB images) and 3D (RGB-D) approaches were tested and compared, providing promising results and perspectives in terms of the potential application of these technologies at the vineyard scale (in situ yield estimation). Another interesting use in viticulture was presented in the research of Palacios et al. [5], where they combined computer vision (RGB images) and machine learning for assessing cluster compactness (degree of the aggregation of its berries) under field conditions (system mounted on an all-terrain vehicle). In this study, the bunches were detected and classified to perform the cluster compactness determination using a Gaussian process regression model. The authors highlighted the potential applicability of this method to determine the spatial variability of cluster compactness in commercial vineyards. As was stated by Palacios et al. [5] and Hacking et al. [4], fruit detection is the first mandatory step to perform other calculations. In this regard, Zemmour et al. [2], presented an automatic parameter-tuning procedure for fruit detection. They developed a tuning process to determine the best fit values of the algorithm parameters to enable easy adaption to different kinds of fruits (shapes, colors) and environments (illumination conditions). In this study, the algorithm was tested under challenging conditions in three crops: red apples, green grapes, and sweet yellow peppers. The algorithm presented successfully detected apples and peppers in variable lighting conditions; however, for green grapes, the authors indicated that there is the need to incorporate some additional features such as morphological parameters to improve the detection process. Estimations of the amount of fruit are important for yield predictions, but also for the right moment to harvest them [4]. The study presented by Valente et al. [3] explored the use of a small-sized electrochemical sensor mounted on a UAV for sensing ethylene concentration in an apple orchard. The latter was the first study focused on investigating the feasibility of ethylene-sensitive sensors in a fruit orchard. However, the results are not conclusive for harvest decisions (fruit maturity). This study opens a research area in this field.

As RGB and RGB-D information, temperature is another variable that can be remotely measured to detect some plant conditions, such as water status and stresses (biotic and abiotic). New technologies of infrared sensors/cameras and computational analysis have allowed a faster and accurate characterization of canopy temperatures. Romero-Bravo et al. [10] presented an application of thermography for estimating grain yield and carbon isotope discrimination in wheat genotypes growing under water stress and full irrigation conditions. The results of this study show that the water regime influences the thermal approach, showing better results under water stress conditions. The authors highlighted that more complex models are needed to estimate grain yield and carbon isotopes since the environmental conditions have a strong influence on the temperature profile of the plants.

Bushfires are one of the climatic anomalies that have increased in number, severity, and window of opportunity within agricultural seasons. For grapevines, they present a critical problem due to smoke contamination and smoke taint. Fuentes et al. [7] proposed the first artificial intelligence approach to model smoke contamination in canopies and smoke taint in grapes and wines using non-invasive infrared thermal imagery (IRTI) and near-infrared spectroscopy (NIR), producing highly accurate machine learning models. From the same research group, further applications of remote sensing and machine learning modelling rendered one of the first specific models to assess aroma profiles of cocoa beans for chocolate manufacturing based on canopy architecture profiles at harvest [6]. These two technological developments can assist growers in combatting environmental hazards and predict quality traits of final products.

Mechanization and agricultural management practices can require significant labor and investment that may not necessarily secure efficiency. Mechanical harvesting can be considered a hot topic in agriculture that requires technology to monitor different aspects to increase productivity. A vibration monitoring system for citrus harvesting was proposed and tested to improve fruit detachment frequency with promising results [8]. Other management practices such as pesticide application require accurate monitoring methods to assess efficiency in the distribution of droplets within crop canopies to minimize detrimental effects in the environment and maximize application efficiency. A rapid method to detect spraying deposit was developed based on capacitance sensors [13].

Agriculture involves not only crop production but also animal farming. Digital technologies have been applied in recent years to monitor the quality of animal feed and to detect animals in order to extract information from remote sensing systems that can provide information on physiological stresses and the general welfare of the animals. One paper researched the use of NIR to predict forage quality of warm-season legumes using machine learning modelling with high accuracy [9]. For animals, a different region of interest from pig bodies was successfully detected using convolutional network deep learning techniques, which may allow for more efficient extraction of information from animals to identify biotic or abiotic stress-related problems [12].

The diversity in applications within this Special Issue makes evident the importance of novel research on new and emerging technologies for the agricultural industry.

## References

[1] Rueda-Ayala V.P., Peña J.M., Höglind M., Bengochea-Guevara J.M., Andújar D. (2019). Comparing UAV-based technologies and RGB-D reconstruction methods for plant height and biomass monitoring on grass ley. Sensors.

[2] Zemmour E., Kurtser P., Edan Y. (2019). Automatic parameter tuning for adaptive thresholding in fruit detection. Sensors.

[3] Valente J., Almeida R., Kooistra L. (2019). A Comprehensive Study of the Potential Application of Flying Ethylene-Sensitive Sensors for Ripeness Detection in Apple Orchards. Sensors.

[4] Hacking C., Poona N., Manzan N., Poblete-Echeverría C. (2019). Investigating 2-d and 3-d proximal remote sensing techniques for vineyard yield estimation. Sensors.

[5] Palacios F., Diago M.P., Tardaguila J. (2019). A Non-Invasive Method Based on Computer Vision for Grapevine Cluster Compactness Assessment Using a Mobile Sensing Platform under Field Conditions. Sensors.

[6] Fuentes S., Chacon G., Torrico D.D., Zarate A., Gonzalez Viejo C. (2019). Spatial variability of aroma profiles of cocoa trees obtained through computer vision and machine learning modelling: A cover photography and high spatial remote sensing application. Sensors.

[7] Fuentes S., Tongson E.J., De Bei R., Gonzalez Viejo C., Ristic R., Tyerman S., Wilkinson K. (2019). Non-invasive tools to detect smoke contamination in grapevine canopies, berries and wine: A remote sensing and machine learning modeling approach. Sensors.

[8] Castro-Garcia S., Aragon-Rodriguez F., Sola-Guirado R.R., Serrano A.J., Soria-Olivas E., Gil-Ribes J.A. (2019). Vibration Monitoring of the Mechanical Harvesting of Citrus to Improve Fruit Detachment Efficiency. Sensors.

[9] Baath G.S., Baath H.K., Gowda P.H., Thomas J.P., Northup B.K., Rao S.C., Singh H. (2020). Predicting Forage Quality of Warm-Season Legumes by Near Infrared Spectroscopy Coupled with Machine Learning Techniques. Sensors.

[10] Romero-Bravo S., Méndez-Espinoza A.M., Garriga M., Estrada F., Escobar A., González-Martinez L., Poblete-Echeverría C., Sepulveda D., Matus I., Castillo D. (2019). Thermal imaging reliability for estimating grain yield and carbon isotope discrimination in wheat genotypes: Importance of the environmental conditions. Sensors.

[11] Yao L., Wang Q., Yang J., Zhang Y., Zhu Y., Cao W., Ni J. (2019). UAV-borne dual-band sensor method for monitoring physiological crop status. Sensors.

[12] Psota E.T., Mittek M., Pérez L.C., Schmidt T., Mote B. (2019). Multi-pig part detection and association with a fully-convolutional network. Sensors.

[13] Wang P., Yu W., Ou M., Gong C., Jia W. (2019). Monitoring of the pesticide droplet deposition with a novel capacitance sensor. Sensors.

